# Supplementary material for: From sequence to enzyme mechanism using multi-label machine learning
Source: BMC Bioinformatics. 2014 May 19;15:150. doi: 10.1186/1471-2105-15-150 (PMC4229970; doi:10.1186/1471-2105-15-150)
Supplement: Additional file 2 — Java code of ml2db. Additional file ml2db_code.tar.gz contains the Java source code to run the multi-label machine learning experiments and save the results to database. The code’s Javadoc is included. [file 1471-2105-15-150-S2.zip › additional file 2/ml2db/utils/doc/uk/ac/standrews/utils/main/guiutils/CommandLineMenu.html]

CommandLineMenu


JavaScript is disabled on your browser.


- Overview
- Package
- Class
- Use
- Tree
- Deprecated
- Index
- Help

- Prev Class
- Next Class

- Frames
- No Frames

- All Classes

- Summary:
- Nested |
- Field |
- Constr |
- Method

- Detail:
- Field |
- Constr |
- Method


uk.ac.standrews.utils.main.guiutils

## Class CommandLineMenu

- java.lang.Object
- - uk.ac.standrews.utils.main.guiutils.CommandLineMenu

- ---

    

  ```
  public class CommandLineMenu
  extends java.lang.Object
  ```

  Stores the options for a command line program.

  Version:
  :   14 Dec 2007

  Author:
  :   Luna De Ferrari luna.deferrari-at-ed.ac.uk

- - ### Constructor Summary

    Constructors

    Constructor and Description

    `CommandLineMenu(java.lang.String name,
    java.lang.String programDescription,
    java.util.ArrayList<CommandOption> options,
    java.lang.String exampleCommand,
    java.lang.String authors)`
  - ### Method Summary

    Methods

    | Modifier and Type | Method and Description |
    | --- | --- |
    | `java.lang.String` | `toString()` "-n  The file containing the network e.g. |

    - ### Methods inherited from class java.lang.Object

      `equals, getClass, hashCode, notify, notifyAll, wait, wait, wait`

- - ### Constructor Detail


    - #### CommandLineMenu

      ```
      public CommandLineMenu(java.lang.String name,
                     java.lang.String programDescription,
                     java.util.ArrayList<CommandOption> options,
                     java.lang.String exampleCommand,
                     java.lang.String authors)
      ```
  - ### Method Detail


    - #### toString

      ```
      public java.lang.String toString()
      ```

      "-n  The file containing the network e.g. in sif format (any Cytoscape format is valid http://www.cytoscape.org/)"
      + "\n" +
      "-o  File containing the node names to act as origin for the path search (one per line)"
      + "\n" +
      "-t  File containing the node names to act as targets for the path search (one per line)"
      + "\n" +
      "-r  The path and name of the report file to be created"
      + "\n" +
      "-s  e.g. \\s for space \\t for tab or , (comma). Default is tab separated."
      + "\n" +
      "-m method (algorithm) Either 0 = single shortest path (directed and undirected) or 1 = PESCA multi shortest path (all undirected shortest paths). Default is 0."
      + "\n" +
      "-a  (optional) The directory containing the .NA (node attributes) .EA (edge attributes) Cytoscape files."
      + "\n" + "-h > gets this help."+ "\n" +

      **Overrides:**
      :   `toString` in class `java.lang.Object`


- Overview
- Package
- Class
- Use
- Tree
- Deprecated
- Index
- Help

- Prev Class
- Next Class

- Frames
- No Frames

- All Classes

- Summary:
- Nested |
- Field |
- Constr |
- Method

- Detail:
- Field |
- Constr |
- Method
